# Advances in the multifunctional roles of CX3CL1 in the central nervous system

**DOI:** 10.3389/fnagi.2026.1696689

**Published:** 2026-01-26

**Authors:** Yuhui Chen, Junli Liu, Sen Zhang, Mengke Gao, Fan Wang, Min Cai, Chengbiao Lu, Shaomin Li, Jianhua Zhao

**Affiliations:** 1 Henan Joint International Research Laboratory of Neurorestoratology for Senile Dementia, The First Affiliated Hospital of Xinxiang Medical University, Xinxiang, China; 2 Xinxiang Medical University, Xinxiang, China; 3 Sino-UK Joint Laboratory of Brain Function and Injury of Henan Province, Department of Physiology and Neurobiology, Xinxiang Medical University, Xinxiang, China; 4 Ann Romney Center for Neurologic Diseases, Brigham and Women’s Hospital, Harvard Medical School, Boston, MA, United States

**Keywords:** aging, CNS, cognitive dysfunction, CX3CL1, myelin regeneration, neuroinflammation, synaptic plasticity

## Abstract

C-X3-C motif chemokine ligand 1 (CX3CL1), a structurally unique chemokine in the central nervous system (CNS), shapes physiological and pathological processes via specific binding to its receptor, C-X3-C motif chemokine receptor 1 (CX3CR1). Empirical evidence indicates that this signaling axis exerts dual neuroinflammatory effects: It restrains microglial hyperactivation, yet can promote inflammation under conditions such as chronic stress. Notably, it preserves synaptic plasticity and facilitates remyelination. Age-associated reductions in CX3CL1 exhibit a strong correlation with cognitive decline; administration of exogenous CX3CL1 partially mitigates these deficits. This study provides a comprehensive account of the multifaceted functions and regulatory mechanisms of CX3CL1 in CNS diseases, thereby establishing a basis for potential new therapeutic targets.

## 1 Introduction

**Figure 1:**
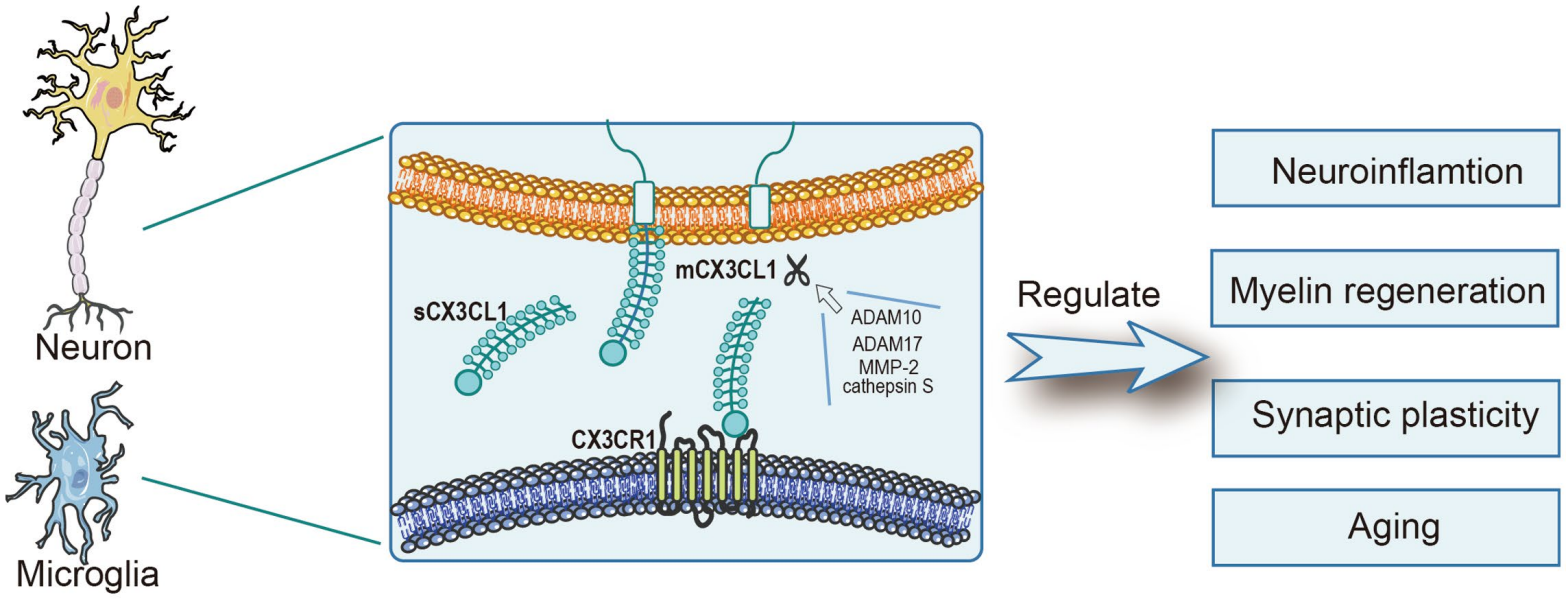
CX3CL1, primarily synthesized by neuronal cells, modulates numerous physiological and pathological processes. Its cleavage is dependent on ADAM10, ADAM17, MMP-2, and cathepsin S under both physiological and pathological conditions. After its interaction with CX3CR1, CX3CL1 partakes in key processes such as neuroinflammation control, myelin regeneration, synaptic plasticity maintenance, and the modulation of aging progression.

C-X3-C motif chemokine ligand 1 (CX3CL1), also known as fractalkine (FKN), is a 373-amino acid (~95 kDa) protein containing an N-terminal chemokine domain, a mucin-like stalk, a transmembrane region, and a short cytoplasmic tail. Its membrane-bound form primarily mediates cellular adhesion, while the secreted isoform (soluble CX3CL1,sCX3CL1) potently recruits monocytes and natural killer (NK) cells (Eugenin et al., 2023; Winter et al., 2020). Under physiological conditions, a disintegrin and metalloproteinase 10 (ADAM10) mediates the cleavage of membrane-anchored CX3CL1, thereby generating its soluble form, and this process is critical for maintaining functional homeostasis (Gunner et al., 2019). However, during inflammation or stress, additional proteases, including ADAM17, matrix metalloproteinase-2 (MMP-2), and cathepsin S, are involved in sCX3CL1 production (Bourd-Boittin et al., 2009; Clark et al., 2009; Garton et al., 2001; Tarozzo et al., 2003). Both isoforms of CX3CL1 bind exclusively to C-X3-C motif chemokine receptor 1 (CX3CR1) and modulate microglial activity via downstream signaling cascades (Harrison et al., 1998; Tarozzo et al., 2003; Tarozzo et al., 2002). Notably, CX3CL1 exerts protective effects in various central nervous system (CNS) diseases: In neuroinflammatory models, it inhibits multiple inflammatory pathways to reduce the release of neuroinflammatory factors (Ge et al., 2022); in the cuprizone mouse model of demyelination, it drives both the replicative expansion and the functional specialization of oligodendrocyte precursor cells (OPCs) (de Almeida et al., 2023); in synaptic injury models, it promotes synaptic repair by reducing inflammatory factor levels and enhancing neurotrophic factor release (Li et al., 2018); and in age-related cognitive impairment models, it upregulates the expression of neurotrophic factors to reverse cognitive dysfunction in aged mice (Takei et al., 2022). Nevertheless, CX3CL1 exhibits context-dependent complexity: Despite its general protective role, it occasionally exerts pro-inflammatory or neurotoxic effects (Lauro et al., 2015). These findings lay the groundwork for exploring its multi-dimensional CNS functions. Overall, the CX3CL1 axis is indispensable for the regulation of neuroinflammation, remyelination, synaptic plasticity, aging, and cognition, maintaining neural homeostasis and fine-regulating neurological disease progression (Cardoso et al., 2018; de Almeida et al., 2023; Kang et al., 2025; Subbarayan et al., 2022) (Figure 1). In this review, we identify CX3CL1 as a core regulatory hub that advances both basic neurobiological research and the precision treatment of neurological disorders, with the ultimate goal of translating mechanistic insights into tangible clinical benefits.

## 2 CX3CL1 and neuroinflammation

CX3CL1 acts as a pivotal regulator of neuroinflammatory processes in the CNS (Gutierrez et al., 2025), and it is mainly accomplished by regulating neuroglial cell activity. Microglia, in particular, serve as key mediators in neuroinflammatory processes (Luchena et al., 2022; Shao et al., 2022). In physiological settings, when CX3CL1 binds to CX3CR1 on microglia, it maintains microglia in a quiescent state (De Felice et al., 2022; Wood and Singer, 2025). During pathological conditions, CX3CL1 deficiency drives microglial polarization toward a pro-inflammatory (M1) phenotype, with elevated inducible nitric oxide synthase (iNOS) activity observed concurrently, which further exacerbates neuroinflammatory responses (Li et al., 2018). Exogenous CX3CL1 promotes microglial polarization toward an anti-inflammatory (M2) phenotype (marked by arginase 1 (Arg1) upregulation), alleviating inflammation (Jiang et al., 2022). Therefore, CX3CL1 exerts its central regulatory role by driving microglial phenotype switching.

The interaction between CX3CL1 and CX3CR1 forms a complex regulatory system that impacts both the production of inflammatory mediators and neuronal viability (Goshi et al., 2020). Donohue et al. observed that, compared to individuals with mild or moderate ischemic stroke and healthy controls, patients with severe ischemic stroke exhibited significantly lower plasma CX3CL1 levels, and these levels were inversely associated with systemic inflammation markers and.

**Figure 2:**
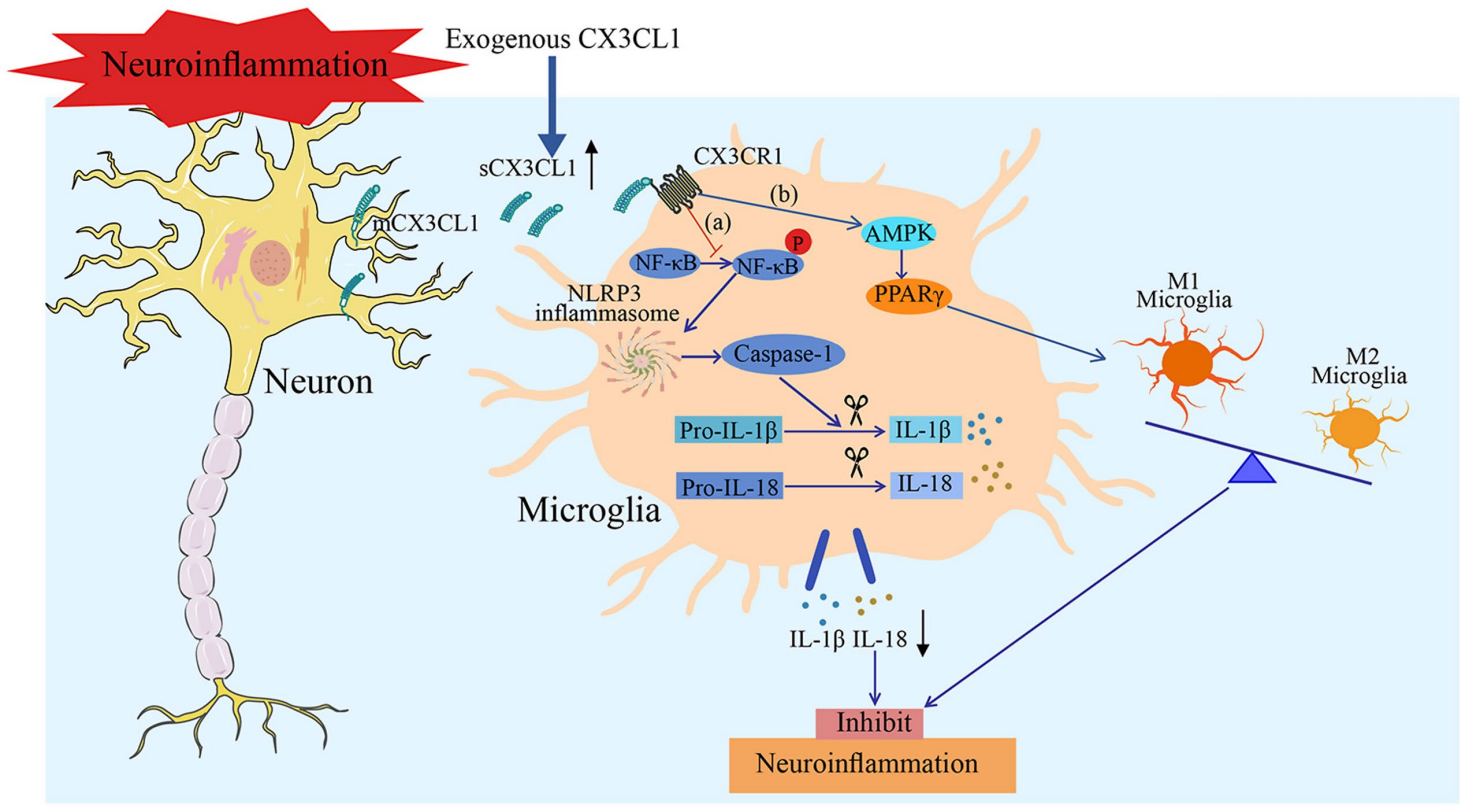
The potential mechanisms through which CX3CL1 alleviates neuroinflammation include: **(a) I**mpeding NLRP3 inflammasome and NF-κB pathway activation to reduce the secretion of inflammatory factors and **(b)** Activating the AMPK/PPARγ pathway to promote M2 polarization of microglia, thus mitigating neuroinflammation and improving neurological function.

Poorer outcomes at the 180-day follow-up (Donohue et al., 2012). After middle cerebral artery occlusion and reperfusion (MCAO/R), CX3CL1 levels are markedly reduced. However, intracerebral supplementation of exogenous CX3CL1 inhibits NLRP3 inflammasome priming and activation, suppresses NF-κB signaling cascade activation, decreases concentrations of interleukin-1β (IL-1β) and IL-18, reduces infarct size, and ameliorates neurological deficits (Ge et al., 2022). Within the permanent middle cerebral artery occlusion (pMCAO) model, CX3CL1 enhances the activity of the adenosine A3 receptor (A3R), which further curtails inflammatory cytokines and impairs the progressive escalation of inflammation in the ischemic penumbra (Rosito et al., 2014). In intracerebral hemorrhage models, CX3CL1 drives microglial polarization toward the M2 phenotype through the AMP-activated protein kinase (AMPK)/peroxisome proliferator-activated receptor *γ* (PPARγ) pathway, thereby reducing neuroinflammation, accelerating hematoma clearance, and improving neurological function (Chen et al., 2023; You et al., 2022) (Figure 2). In models related to stroke rehabilitation, treadmill exercise increases CX3CL1 and its receptor CX3CR1, which promotes neurogenesis in the subventricular zone (SVZ) and dentate gyrus (DG) while controlling inflammation and enhancing synaptic plasticity (Ge et al., 2025). In 1-methyl-4-phenyl-1,2,3,6-tetrahydropyridine (MPTP)-induced Parkinson’s disease (PD) models, intranigral stereotaxic delivery of CX3CL1-overexpressing adeno-associated virus (AAV) in mice reduces the release of pro-inflammatory cytokines such as tumor necrosis factor-*α* (TNF-α) and IL-1β, alleviates dopaminergic neuronal loss, and improves motor function (Angelopoulou et al., 2020; Morganti et al., 2012). In the amyotrophic lateral sclerosis (ALS) model induced by overexpression of the mutant human Cu/Zn superoxide dismutase 1 (SOD1) gene carrying the Gly93Ala substitution, dysregulation of the CX3CL1/CX3CR1 axis disrupts the homeostatic balance of microglial polarization: CX3CL1 exerts neuroprotective effects by modulating the M1/M2 polarization balance in the early stage, yet subsequent downregulation of its expression exacerbates inflammatory dysregulation and accelerates the pathological progression of the disease (Sarikidi et al., 2022; Zhang et al., 2018). In hypothalamic inflammation induced by a high-fat diet (HFD), exogenous CX3CL1 inhibits inflammation and reduces overeating through the brain-derived neurotrophic factor (BDNF)/tyrosine receptor kinase B (TrkB) pathway (Banerjee et al., 2022; Kawamura et al., 2022). A separate study demonstrated that silymarin increases CX3CL1 levels in the cerebral cortex of obese mice with cerebral ischemia, enhances post-ischemia survival, and highlights CX3CL1’s role in promoting recovery after stroke (Rodriguez-Cortes et al., 2024).

CX3CL1 is primarily anti-inflammatory, yet it can exert pro-inflammatory effects under certain pathological conditions (Liu et al., 2020; Zhang et al., 2021). For example, in chronic unpredictable stress (CUS) models, CX3CL1 overexpression sharply increases reactive oxygen species (ROS) levels, triggers neuronal apoptosis and dysfunction, and impairs synaptic plasticity (Liu et al., 2020; Qin et al., 2023). Nevertheless, CX3CL1 regulates neuroinflammation through a dual regulatory mode via multiple pathways (Table 1). Studies have confirmed that this molecule can directly modulate microglial activation and phenotypic switching (Camacho-Hernandez and Pena-Ortega, 2023) and indirectly regulate neuroinflammatory responses by inhibiting or promoting monocyte infiltration into the brain (Bai et al., 2023). Therefore, CX3CL1 is not only a key molecular hub mediating transcellular communication between neurons and microglia but also a core regulator of the neuroinflammatory network through bidirectional regulatory mechanisms in complex pathological microenvironments. This feature further underscores the clinical value and scientific significance of developing therapeutic strategies for related neurological disorders by targeting the CX3CL1 signaling pathway.

**Table 1:** The function of CX3CL1 in neuroinflammation.

CX3CL1 Function	Model	Reference
Predicting Stroke Prognosis	Severely ischemic stroke patients	Donohue et al. (2012)
Impeding NLRP3 inflammasome and NF-κB pathway activation to reduce the secretion of inflammatory factors	MCAO/R	Ge et al. (2022)
Enhancing A3R activity to inhibit inflammatory factors and delay the progression of the ischemic penumbra	pMCAO	Rosito et al. (2014)
Mitigating neuroinflammation and promoting hematoma clearance via the CX3CR1/AMPK/PPARγ pathway	Intracerebral Hemorrhage Model	Chen et al. (2023) and You et al. (2022)
Promoting hippocampal neurogenesis while regulating inflammation and enhancing synaptic plasticity	Models related to stroke rehabilitation	Ge et al. (2025)
Reducing the release of proinflammatory cytokines, alleviating the loss of dopaminergic neurons, and improving motor function	PD	Angelopoulou et al. (2020) and Morganti et al. (2012)
Providing neuroprotective effects by regulating the M1/M2 balance	ALS	Sarikidi et al. (2022) and Zhang et al. (2018)
Ameliorating neural damage through the BDNF/TrkB pathway	Hypothalamic inflammation	Banerjee et al. (2022) and Kawamura et al. (2022)
Increasing ROS levels, leading to neuronal apoptosis and functional dysfunction	CUS	Liu et al. (2020) and Qin et al. (2023)

## 3 CX3CL1 and myelin regeneration

Myelin regeneration, a key repair mechanism in the CNS involving intricate interactive processes, proves vital because it sheathes neuronal axons: It not only accelerates nerve impulse transmission via saltatory conduction but also actively regulates neural function (Osso and Hughes, 2024). Recent research has increasingly highlighted the regulatory role of the chemokine CX3CL1 in this regenerative process (Mendiola et al., 2022; O'Sullivan and Dev, 2017; Schroder et al., 2023; Watson et al., 2021).

**Figure 3:**
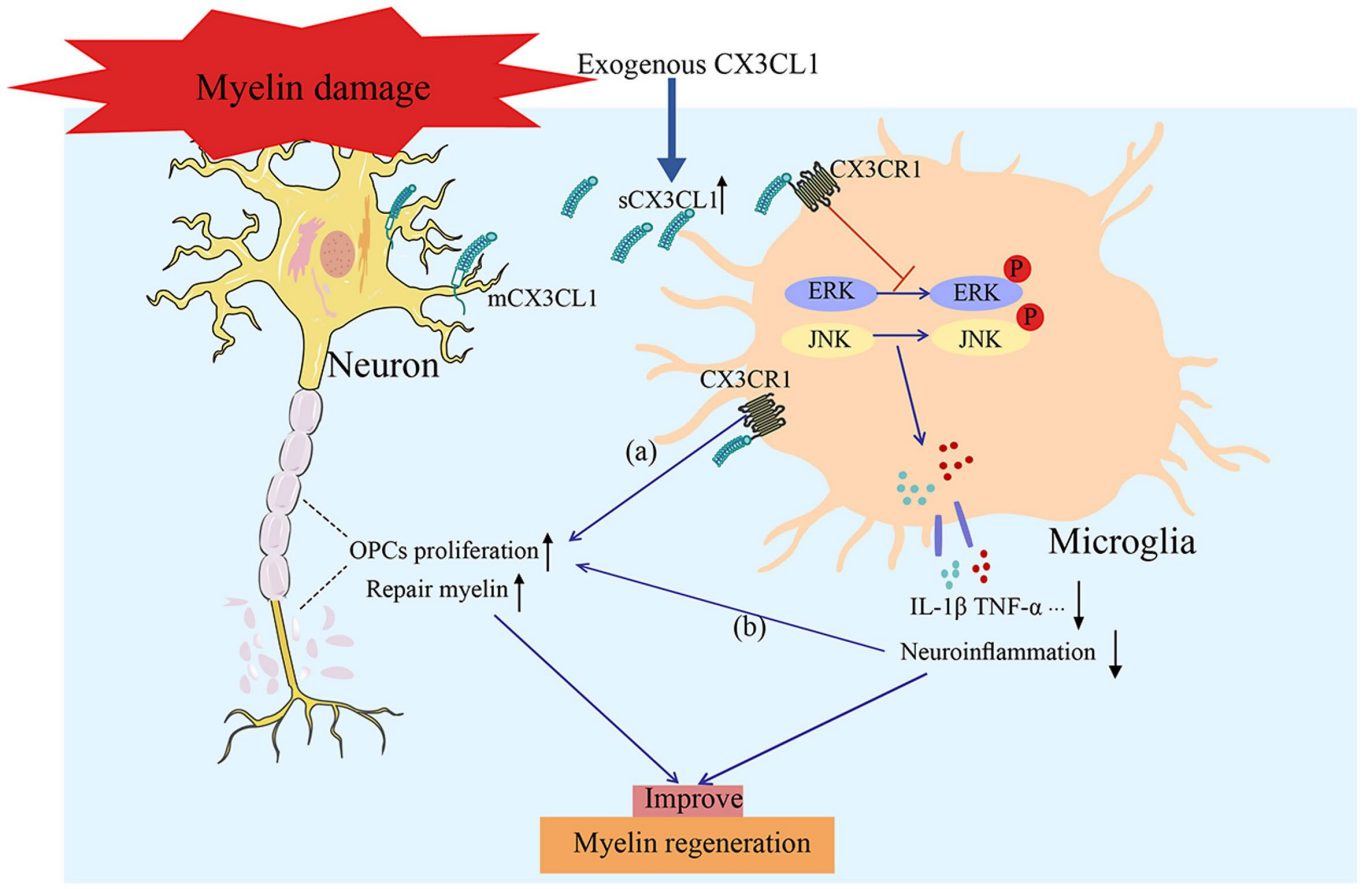
CX3CL1 promotes remyelination via dual pathways: **(a)** Directly stimulating OPCs to proliferate and differentiate; and **(b)** suppressing neuroinflammation and improving the microenvironment (including promoting microglial M2 polarization/proliferation and downregulating IL-1β/TNF-*α*), holding promise as a potential therapeutic target for myelin repair.

OPCs serve as the primary cellular source for myelin regeneration, and their activities are crucially regulated by CX3CL1. The binding of this molecule to CX3CR1 has been demonstrated to robustly enhance OPC proliferation and differentiation. In line with this, intracerebroventricular administration of CX3CL1 increases the number of newly generated OPCs in the SVZ and promotes their maturation (Watson et al., 2021); moreover, it indirectly maintains the differentiation potential of OPCs by restraining the inflammatory activation of microglia in the microenvironment (de Almeida et al., 2023). In chronic cerebral hypoperfusion models, exercise-induced upregulation of CX3CL1 reduces phosphorylation of the extracellular signal-regulated kinase (ERK)/c-Jun N-terminal kinase (JNK) (Jiang et al., 2017). This downregulation suppresses neuroinflammation, promotes microglial M2 polarization, and alleviates OPC differentiation arrest, thereby accelerating remyelination and cognitive recovery. In hydrogen peroxide (H₂O₂)-induced demyelination models, exogenous CX3CL1 attenuates demyelination by upregulating the expression of the myelin basic protein (MBP) while modulating vimentin expression. CX3CL1 reduces lesion severity, enhances astrocyte functions, and underscores its protective role against oxidative stress-associated demyelination (O'Sullivan and Dev, 2017). In cuprizone-induced chronic demyelination models, CX3CL1 treatment enhances remyelination through microglial repopulation. This shows that it can exert repair effects both directly on myelinating cells and indirectly via microglia-mediated immunoregulation, including downregulation of IL-1β and TNF-*α* (Tahmasebi et al., 2024; Tahmasebi et al., 2021). Owing to this dual mechanism of action, CX3CL1 holds promise as a prospective therapeutic target for myelin restoration (Figure 3). Clinical studies have confirmed that myelin damage-related white matter hyperintensities (WMHs) exhibit a strong correlation with cognitive deterioration across various populations, including cognitively unimpaired individuals, patients with mild cognitive impairment (MCI), stroke survivors, and individuals diagnosed with Alzheimer’s disease (AD). Importantly, this relationship is especially prominent in patients with MCI and post-stroke cohorts, highlighting WMHs as a key risk factor for the onset and progression of cognitive impairment. Given CX3CL1’s critical regulatory role in myelin repair, it is proposed as a promising therapeutic candidate for ameliorating myelin damage-induced cognitive dysfunction, thereby offering a novel strategy for intervention in such cognitive deficits (Li et al., 2023; Roseborough et al., 2023).

## 4 CX3CL1 and synaptic plasticity

**Figure 4:**
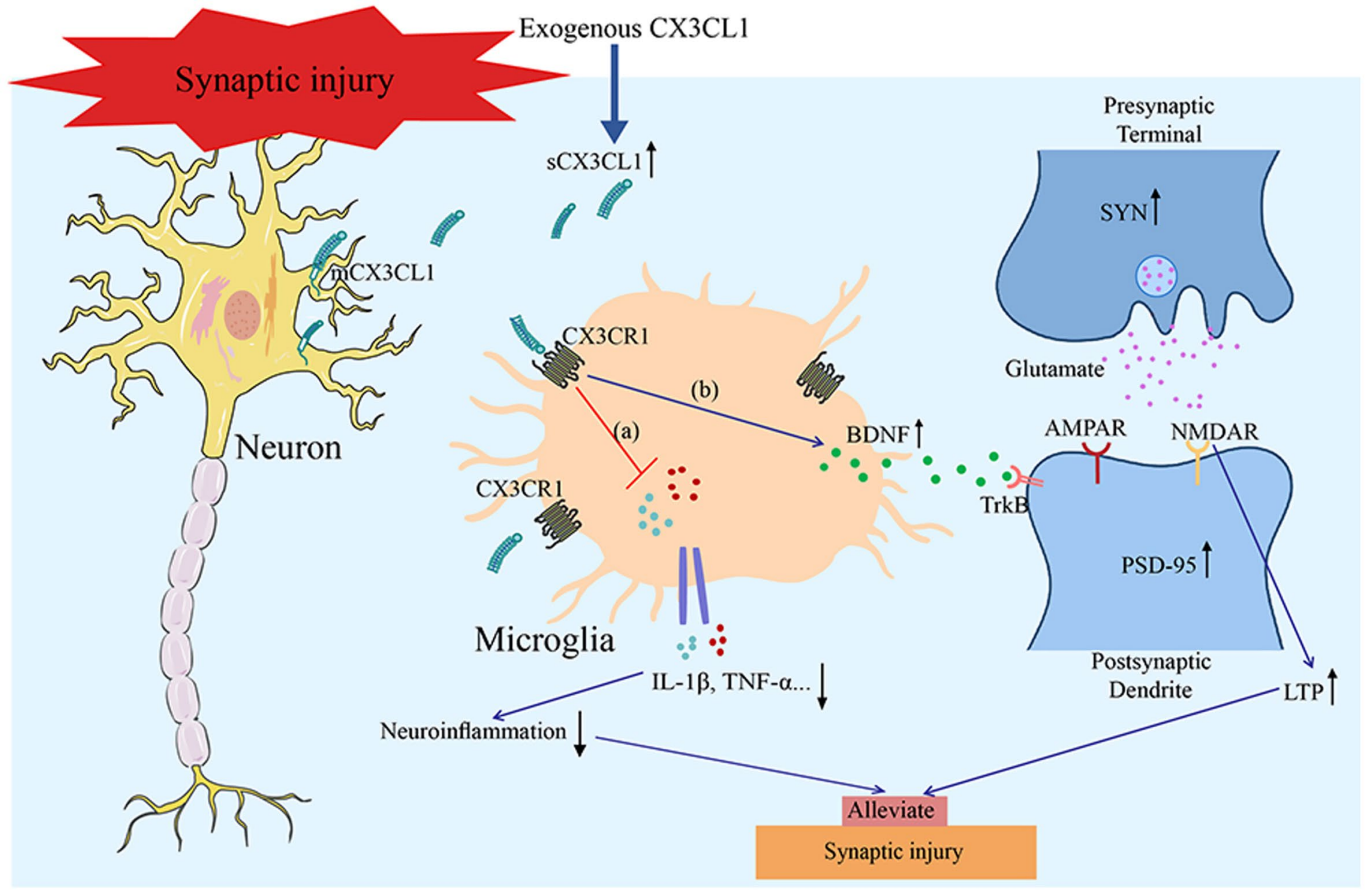
CX3CL1 mitigates synaptic damage and improves synaptic function via dual mechanisms: **(a)** Suppressing neuroinflammation and **(b)** Prompting microglia to produce and release neurotrophic factors, including BDNF. These factors further enhance the expression of synaptic proteins (PSD95 and SYN) to ameliorate synaptic dysfunction.

CX3CL1 also plays a critical regulatory role in synaptic plasticity (Bachstetter et al., 2011; Vukovic et al., 2012). In normal physiological states, the CX3CL1/CX3CR1 axis refines the neurogenic microenvironment by curbing excessive microglial activation (Bachstetter et al., 2011). Studies show that it positively regulates synaptic plasticity in healthy brain regions and maintains synaptic function during development and adulthood via microglia-mediated neuroprotection (Arnoux and Audinat, 2015; Bolos et al., 2018; Brett et al., 2022; Paolicelli et al., 2014). For example, the CX3CL1/CX3CR1 signaling axis facilitates the survival of newborn neurons and augments synaptic activity, thereby optimizing neural network function. It also participates in synaptic pruning, defined as the microglial engulfment of unnecessary or dysfunctional synapses, which is a critical process for learning and memory (Nash et al., 2015). Moreover, CX3CL1 boosts the function of the N-methyl-D-aspartate receptor (NMDAR) and modulates synaptic plasticity through a mechanism that increases adenosine release, thereby activating the NMDAR co-agonist site (Scianni et al., 2013). Under pathological conditions, CX3CL1 is equally critical. In Huntington’s disease (HD) models, its deficiency leads to aberrant localization of postsynaptic density protein 95 (PSD-95) in neurons, while exogenous supplementation restores long-term depression (LTD) in model mice, highlighting its role in maintaining synaptic plasticity through the regulation of neuron–microglia interactions (Kim et al., 2020). In 5-lipoxygenase-deficient (5-LO^−^/^−^) mice, reduced CX3CL1 levels in the motor cortex cause synaptophysin (SYN) abnormalities, disrupting synaptic pruning and exacerbating motor deficits (Barbosa-Silva et al., 2022). In CX3CL1-knockout mice with impaired hippocampal long-term potentiation (LTP), overexpression of sCX3CL1 restores LTP by inhibiting M1 polarization, reducing pro-inflammatory cytokine levels, promoting the proliferation and differentiation of dentate gyrus (DG) OPCs, and enhancing neurogenesis (Winter et al., 2020). In the PS19 transgenic mouse model of AD, CX3CL1 overexpression mitigates abnormal tau aggregation, enhances synaptic density, improves synaptic plasticity, and reverses neuronal loss and cognitive deficits, highlighting this pathway as a potential therapeutic target for ameliorating synaptic dysfunction in AD (Fan et al., 2020). In glutamate-induced hippocampal injury, CX3CL1 activates the extracellular signal-regulated kinase 1/2 (ERK1/2) and phosphatidylinositol 3-kinase/protein kinase B (PI3K/Akt) pathways, thereby inhibiting neuronal apoptosis by reducing glutamate-mediated *α*-amino-3-hydroxy-5-methyl-4-isoxazolepropionic acid receptor (AMPAR) currents and intracellular calcium overload (Limatola et al., 2005). Parkhurst et al. (2013) showed that CX3CL1 mitigates neurotoxicity via NMDAR activation, boosting cAMP response element-binding protein (CREB) phosphorylation and increasing levels of BDNF and TrkB (Lauro et al., 2014; Parkhurst et al., 2013). Taken together, these findings illustrate that CX3CL1 regulates synaptic plasticity through multifaceted interactions with microglia, neurogenic processes, and key signaling pathways. Its regulatory role dynamically adapts to physiological homeostasis and pathological perturbations, providing critical insights for targeting synaptic dysfunction in neurological disorders (Figure 4).

## 5 CX3CL1 and aging

With advancing age, CX3CL1 expression undergoes substantial changes and exhibits a strong association with the pathogenesis and progression of neurodegenerative disorders. It modulates the activity of microglia and maintains the stability of the brain microenvironment, thereby influencing the progression of cognitive deficits and neurodegenerative lesions (Mecca et al., 2018; Wynne et al., 2010). In older humans and animal models, the expression of CX3CL1 typically decreases (Duan et al., 2008; Paolicelli et al., 2014). Wynne et al. demonstrated that in the brains of aged mice, CX3CR1 is persistently downregulated, a change associated with elevated levels of IL-1β and reduced levels of transforming growth factor-β (TGF-β) (Wynne et al., 2010). Harry demonstrated that with aging, the migratory capacity, phagocytic clearance function, and inflammatory regulatory role of microglia are all impaired, and these deficits may stem from a weakened CX3CL1 signaling pathway (Harry, 2013). With advancing age, the gradual decline in the blood–brain barrier (BBB) function makes individuals more susceptible to the influence of neuroinflammation (Banks et al., 2021; Lee and Funk, 2023; Shi et al., 2025; Yang et al., 2025). Verite et al. observed that in the APP/PS1 double-transgenic AD mouse model, the level of CX3CL1 within the BBB is significantly reduced, while it is higher in the brain parenchyma. This suggests that such differential expression may influence central immune.

**Figure 5:**
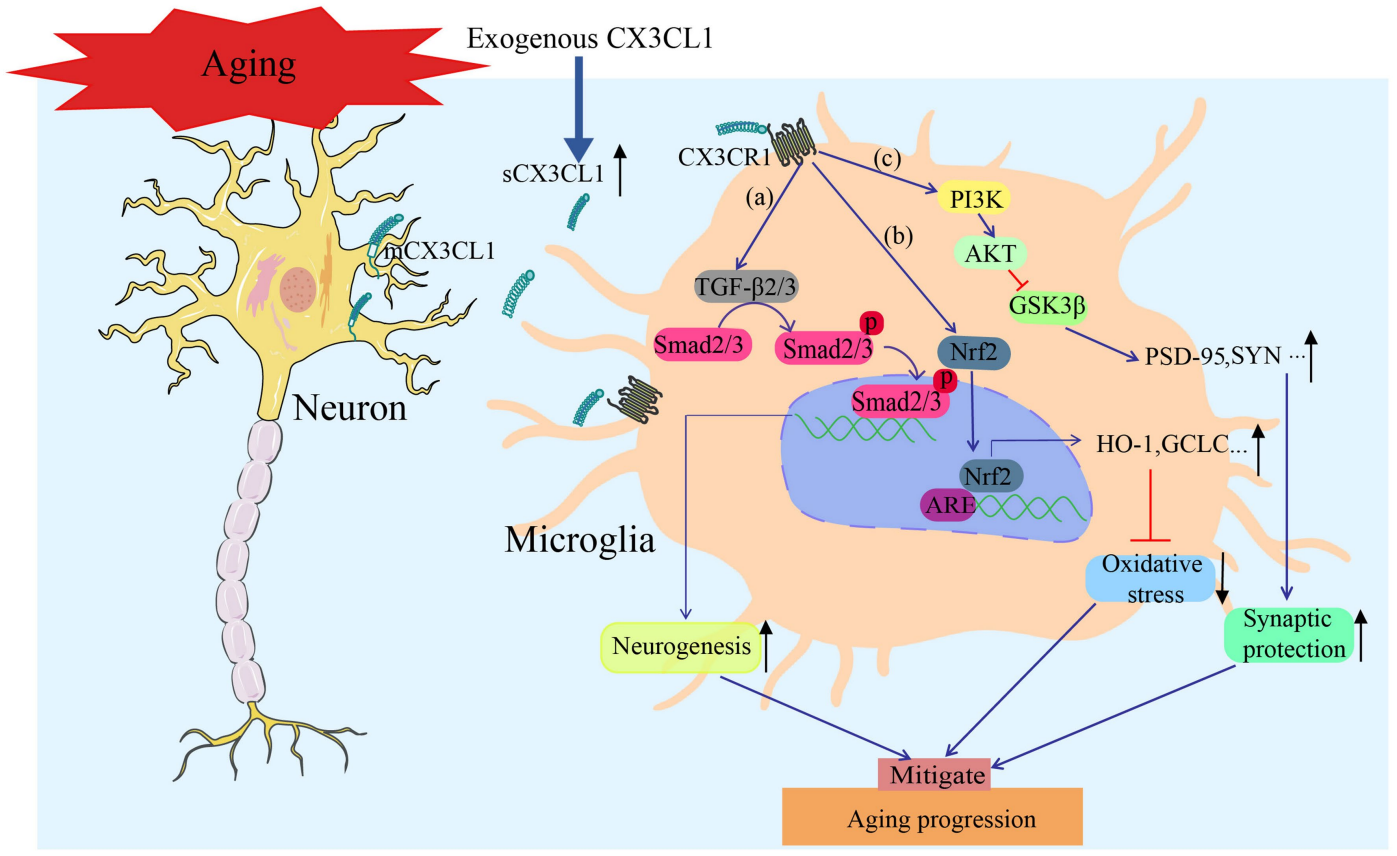
CX3CL1 exerts a protective effect against age-related neurodegeneration via three distinct mechanisms: **(a)** Exerting a pro-neurogenic effect via the TGFβ2/3-Smad2/3 signaling axis to replenish newly generated neurons in aging brain regions; **(b)** activating the Nrf2 pathway to enhance antioxidant capacity, thereby mitigating age-induced oxidative damage to neurons; and **(c)** upregulating synaptic protein expression via the PI3K/AKT/GSK3β pathway to reverse age-associated synaptic dysfunction.

Responses by crossing the BBB (Verite et al., 2018). Building on this, Li et al. further explored the specific functional mechanisms of CX3CL1. They conducted experiments using the 5 **×** FAD mouse model and found that overexpression of the membrane-anchored CX3CL1 fragment significantly reduced amyloid-β (Aβ) deposition in the mouse brain and effectively alleviated neuronal loss. This protective effect is closely related to the intracellular domain of CX3CL1, which can activate the TGF-β2/3-mothers against the decapentaplegic homolog 2/3 (Smad2/3) signaling pathway to enhance neurogenesis, thereby mitigating pathological damage in AD (Fan et al., 2019). Li et al. (2020) successfully delivered CX3CL1 via mesenchymal stem cells (MSCs). In APP/PS1 mice, this delivery approach improved the expression of synapse-related proteins, repaired age-associated synaptic dysfunction, and alleviated memory impairment through the PI3K/AKT/glycogen synthase kinase 3β (GSK3β) pathway (Li et al., 2020). The dynamic role and regulatory mechanisms of CX3CL1 observed in aging and AD animal models have been partially validated in human AD clinical studies, showing stage-specific characteristics that are more aligned with the actual disease progression. In clinical studies on cerebrospinal fluid (CSF) CX3CL1 levels in patients with AD, Perea et al. found that this marker was significantly lower in AD dementia patients compared to healthy individuals (Perea et al., 2018), whereas Bivona et al. observed notably higher CX3CL1 levels in AD patients compared to individuals diagnosed with non-AD dementia (Bivona et al., 2022). These seemingly contradictory findings stem from the different AD stages of the included patients. Perea et al. focused on late-stage AD (dementia phase) with massive neuronal loss, and since CX3CL1 is mainly neuron-derived, its levels drop sharply. In contrast, Bivona et al. enrolled early-stage AD patients with minimal neuronal damage, in which CX3CL1 is upregulated as a “compensatory anti-inflammatory factor” to mitigate neuroinflammation by inhibiting excessive microglial activation and maintaining brain microenvironment homeostasis. Xu et al. recently confirmed this stage-dependent feature in their review: In early AD, the CX3CL1/CX3CR1 signaling pathway is activated, significantly enhancing microglia’s phagocytic clearance of Aβ. However, in late AD, extensive neuronal death causes insufficient CX3CL1 synthesis and secretion, weakening its regulation of microglia, reducing neuroprotective efficacy, and ultimately exacerbating pathological damage (Xu et al., 2025). Gupta et al. further revealed that the profile of CX3CL1 expression in PD cases shows a temporal pattern similar to that observed in AD cases (Gupta et al., 2022). It should be emphasized that the functional role of CX3CL1 in aging is closely linked to oxidative stress. Studies have confirmed that CX3CL1 activates the nuclear factor erythroid 2-related factor 2 (Nrf2) pathway, promoting Nrf2 binding to the antioxidant response element (ARE). This activation subsequently upregulates antioxidant proteins, such as heme oxygenase 1 (HO-1) and glutamate-cysteine ligase catalytic (GCLC) subunit, thereby alleviating oxidative stress-mediated neuronal damage and exerting a key aging-related neuroprotective effect (Lastres-Becker et al., 2014). At the same time, age-driven gut dysbiosis allows neurotoxic gut metabolites to trigger systemic inflammation via the gut–brain axis, impairing BBB integrity and provoking neuroinflammation (Mou et al., 2022). Crucially, CX3CL1 modulates neuroinflammation by controlling microglial polarization, highlighting its potential to mitigate BBB injury via anti-inflammatory mechanisms and thereby slow aging-related progression. To conclude, CX3CL1 modulates microglial activity, preserves the integrity and homeostasis of the BBB, and coordinates the gut–brain axis immune response while activating antioxidant signaling pathways to alleviate oxidative stress-induced damage. With its diverse biological activities and multifaceted regulatory mechanisms, CX3CL1 provides a strong theoretical foundation and promising application potential for the exploration and development of cutting-edge therapeutic strategies targeting age-related neurological diseases (Figure 5).

## 6 CX3CL1 and cognitive function

**Figure 6:**
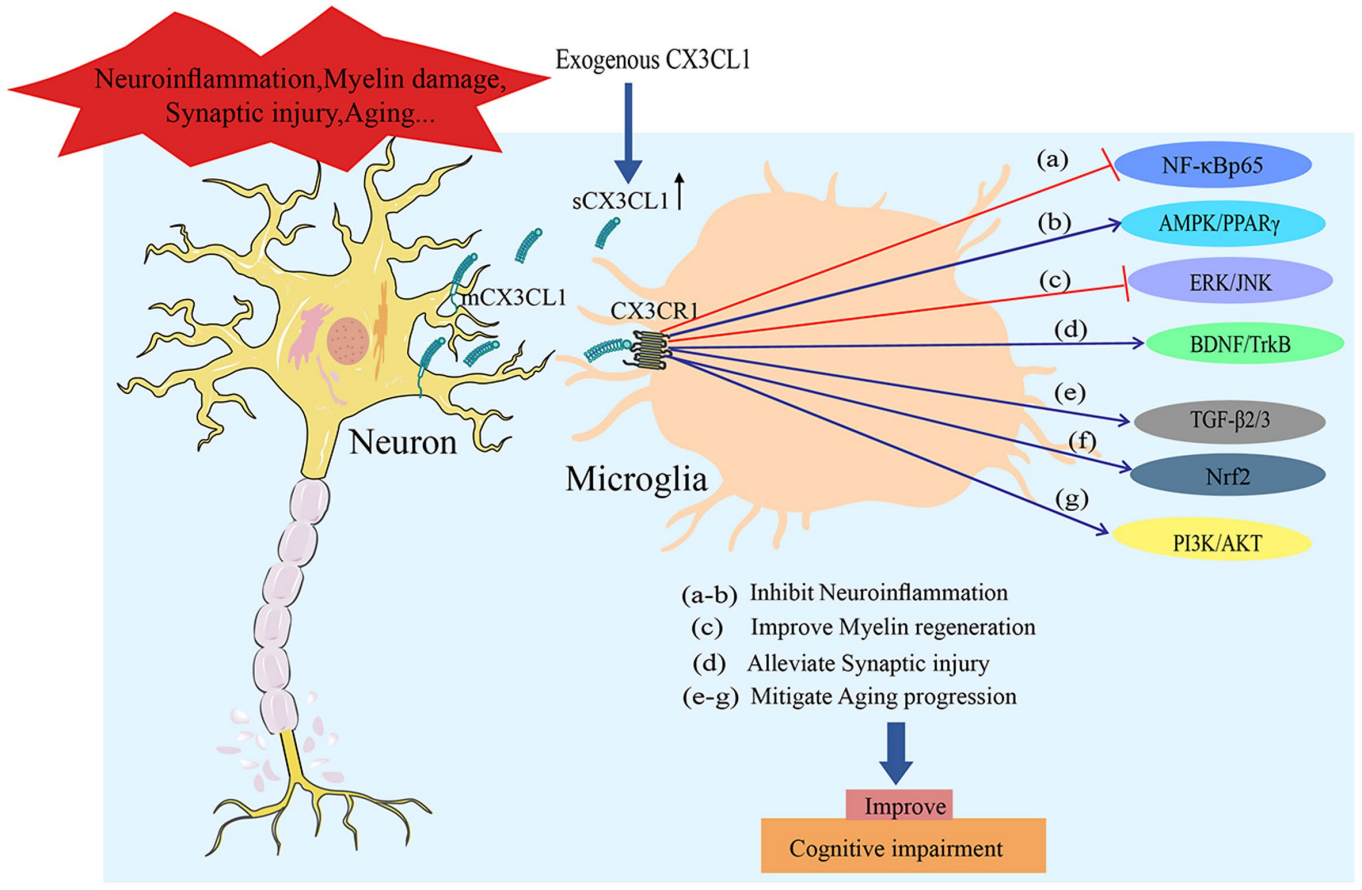
Exogenous CX3CL1 supplementation exerts multi-pathway effects to ameliorate cognitive impairment, with its potential mechanisms summarized as follows. Under pathological conditions such as neuroinflammation, myelin damage, synaptic injury, and aging, exogenous CX3CL1 binds to CX3CR1 on the microglial surface, thereby effectively elevating the level of sCX3CL1. Subsequent actions are mediated via the following targeted pathways: **(a,b)** Inhibition of neuroinflammation through NF-κB pathway blockade and AMPK/PPARγ pathway activation; **(c)** promotion of oligodendrocyte precursor cell proliferation via the ERK/JNK pathway to mediate myelin regeneration; **(d)** alleviation of synaptic injury by activating the BDNF/TrkB pathway; **(e–g)** and delay of the aging process through the activation of the TGF-β2/3, Nrf2, and PI3K/AKT pathways.

In conditions associated with cognitive decline, CX3CL1 shows notable changes in expression and function. Zhou et al. found that its levels are higher in the blood of individuals with MCI but lower in AD patients, highlighting its association with disease stage (Zhou et al., 2023). CX3CL1 affects cognition through multiple pathways, primarily by regulating neuroinflammation. Activated CX3CL1/CX3CR1 signaling suppresses excessive microglial activation, protects neural function, and alleviates cognitive deficits (Ge et al., 2022). Myelin damage disrupts its normal structure and function, impairing cognition. Microglia play a central role in maintaining myelin health and ensuring normal cognitive function (Mcnamara et al., 2023; Wolf et al., 2021; Yi et al., 2024). Qiu et al. (2021) also confirmed that myelin injury activates disease-associated microglia and astrocytes, increasing the incidence of cognitive dysfunction. In this cascade, CX3CL1 modulates microglial activation, reduces the release of inflammatory factors, and augments the regenerative capacity of oligodendrocytes to promote remyelination, highlighting it as a viable therapeutic candidate for cognitive deficits linked to demyelination (de Almeida et al., 2023). In synaptic damage models, CX3CL1 reduces the release of pro-inflammatory factors, prevents the loss of DG calcium-binding proteins, improves synaptic plasticity, and alleviates memory deficits (Cho et al., 2011). Takei et al. (2022) demonstrated that supplementation with exogenous CX3CL1 boosts the population of type 2 neural stem cells, elevates BDNF expression levels, and alleviates cognitive impairment in aged murine models. Sheridan et al. (2014) found that hippocampal CX3CL1 protein levels increase within 2 h after the Morris water maze task and that LTP-inducing stimulation upregulates its expression in the DG. Based on these mechanisms, we can infer that CX3CL1 ameliorates cognitive impairments resulting from neuroinflammation, myelin damage, synaptic injury, aging, and other related conditions (Figure 6).

## 7 Summary and outlook

In the CNS, CX3CL1 regulates neuron–microglia interactions by modulating neuroinflammation. It promotes myelin regeneration, maintains synaptic stability, delays aging, and mitigates cognitive decline under pathological conditions. However, its potential pro-inflammatory effects in chronic stress or specific microenvironments require further investigation. CNS diseases involve complex mechanisms, and single-target interventions show limited efficacy. Therefore, multi-target combination therapy has emerged as a central strategy for the development of CX3CL1-targeted interventions. Combining CX3CL1-based approaches with other neuroprotective or anti-inflammatory drugs is expected to yield synergistic effects. For instance, in AD and PD, co-administration of CX3CL1-targeted therapies with anti-amyloid or anticholinergic agents allows for simultaneous modulation of multiple pathological pathways. Although CX3CL1 holds broad therapeutic potential in CNS disease treatment, its clinical translation faces several challenges. Its expression and function exhibit heterogeneity across different diseases and individuals, which may affect biomarker reliability. In addition, the safety and efficacy of targeted therapies must be validated in large-scale clinical trials. Nevertheless, with technological advancements and in-depth research, the application prospects of CX3CL1 in the CNS will become clearer, laying the foundation for developing stage-specific and multi-dimensional therapeutic strategies and further promoting the translation from mechanistic research to clinical application.
